# The novel HSP90 inhibitor AT13387 potentiates radiation effects in squamous cell carcinoma and adenocarcinoma cells

**DOI:** 10.18632/oncotarget.5363

**Published:** 2015-10-06

**Authors:** Diana Spiegelberg, Adrian Dascalu, Anja C. Mortensen, Andris Abramenkovs, Gamze Kuku, Marika Nestor, Bo Stenerlöw

**Affiliations:** ^1^ Department of Immunology, Genetics and Pathology, Uppsala University, Uppsala, Sweden; ^2^ Unit of Otolaryngology and Head and Neck Surgery, Department of Surgical Sciences, Uppsala University, Uppsala, Sweden

**Keywords:** 17-AAG, synergy, DNA repair, EGFR, CD44v6

## Abstract

Overexpression of heat shock protein 90 (HSP90) is associated with increased tumor cell survival and radioresistance. In this study we explored the efficacy of the novel HSP90 inhibitor AT13387 and examined its radiosensitizing effects in combination with gamma-radiation in 2D and 3D structures as well as mice-xenografts. AT13387 induced effective cytotoxic activity and radiosensitized cancer cells in monolayer and tumor spheroid models, where low drug doses triggered significant synergistic effects on cell survival together with radiation. Furthermore, AT13387 treatment resulted in G2/M-phase arrest and significantly reduced the migration capacity. The expression of selected client proteins involved in DNA repair, cell-signaling and cell growth was downregulated *in vitro*, though the expression of most investigated proteins recurred after 8–24 h. These results were confirmed *in vivo* where AT13387 treated tumors displayed effective downregulation of HSP90 and its oncogenic client proteins.

In conclusion, our results demonstrate that AT13387 is a potent new cancer drug and effective radiosensitizer *in vitro* with an excellent *in vivo* efficacy. AT13387 treatment has the potential to improve external beam therapy and radionuclide therapy outcomes and restore treatment efficacy in cancers that are resistant to initial therapeutic regimes.

## INTRODUCTION

Cancer treatments have undergone dramatic improvement during the last century, much due to our increasing molecular understanding of this elusive disease category. This improvement has translated to increased post-treatment life expectancy and quality of life. Radiotherapy is today highly developed and one of the most successful principles of cancer treatment, but there still remains great potential to improve efficacy and selectivity [1]. One potential avenue to increased effect is novel co-treatments with radiosensitizing compounds, preferably selectively targeting the tumor location or cancer cell phenotype [2, 3]. Generally, pathways involved in DNA repair and cell stress response are suitable candidate targets for such co-therapy, given their important role in cell survival upon radiation exposure.

Heat shock proteins play, as the name implies, a crucial role in the heat shock response. However, they are not only induced in response to cellular stress; they are equally as important under non-stress conditions where they establish protein-protein interactions, restore three-dimensional protein structures and help newly synthesized proteins to fold into their correct confirmation. Heat shock protein 90 (HSP90) is an evolutionarily conserved and highly abundant protein, making up about 1%–2% of the whole proteome in cells [4, 5]. So far, more than 200 HSP90 client proteins have been found, involved in all kinds of cellular responses (e.g. cell cycle progression and growth, cell signaling, migration, transcription factors), many of which are activated in malignancy [5–8]. Accordingly, an increased expression level of HSP90 has been found in several hematologic and solid tumors, including squamous cell carcinomas of the head and neck region (HNSCC) [9] and adenocarcinomas [10–13]. This overexpression is associated with increased tumor cell survival, an effect that is probably due to stabilization of oncogenic cell signaling proteins, which ultimately prohibits apoptosis. However, HSP90 activation status does not always correlate with its expression and HSP90 may form complexes, that along with a number of cochaperones, can affect its function [14, 15]. HSP90 client proteins include mutated P53, MEK, FAK, CDK4, PDGFR, VEGFR2, CDK-4, -6, Kit, ERK and AKT. In addition, HSP90 stabilizes proteins that are known to be associated with protection against radiation-induced cell death, like HER2, EGFR, RAF-1 and AKT [16–19]. Stabilization of constitutively activated signaling proteins, like AKT and ERK, promotes uncontrollable growth.

Targeting HSP90 with its broad repertoire of interaction partners could possibly overcome mutations in downstream signaling proteins and shut down several pathways simultaneously; compounds with this characteristic are therefore highly promising enhancers of radiotherapy efficacy, including external beam radiotherapy and radionuclide therapy using immunotargeting of overexpressed cell-surface proteins [20]. The HSP90 targeting drug 17-N-Allylamino-17-demethoxygeldanamycin (17-AAG) is a less toxic and more stable derivative of the benzoquinone ansamycin antibiotic geldanamycin [21, 22]. 17-AAG has been well characterized for cancer treatment in numerous preclinical and clinical studies. Unfortunately, the usage of 17-AAG alone and/or in combination with other drugs is limited due to bad solubility, high hepatotoxicity and the potential to form toxic metabolites [21]. The novel high-affinity HSP90 inhibitor, AT13387 (2,4-dihydroxy-5-isopropyl-phenyl)-[5-(4-methyl-piperazin-1-ylmethyl)-1,3-dihydro-isoindol-2-yl] thanone, l-lactic acid salt) is a non-geldanamycin inhibitor, which is easier to administer and may have less toxicity than 17-AAG [23]. AT13387 is now undergoing clinical trials in phase I and II including patients with prostate carcinomas, refractory gastrointestinal stromal tumors (GIST) and ALK positive lung cancer [24, 25].

Today, the standard treatment of HNSCC patients with advanced-stage locoregional disease is a multidisciplinary approach involving surgery, chemo- and radiation therapy. But the therapy options are often limited due to high toxicity of the chemotherapeutic drugs and adverse effects of the radiotherapy. With exception of HPV positive tumors, the overall 5-year survival rate of HNSCC is less than 50% and has remained relatively unchanged for the past decades [26, 27]. Chemotherapeutic radiosensitizing agents such as 5-Fluorouracil (5-FU), cisplatin and oxaliplatin are standard therapy for metastatic colorectal cancer in combination with radiotherapy, but the development of chemoresistance is often inevitable. Increasing chemoresistance indicates the demand for new treatment options that target squamous cell carcinoma and adenocarcinoma more effectively and with reduced toxicity.

In this study the efficacy of the novel drug AT13387 was assessed *in vitro* and *in vivo*. We chose to examine the squamous cell carcinoma cell lines H314 and A431 as well as the adenocarcinoma cell lines LS174T and HCT116, as models for high incidence cancer types with overexpression of HSP90, like head and neck cancer and colon cancer. Furthermore, this study investigates migration capacity, cell cycle progression and downregulation of important HSP90 client proteins as well as DNA repair efficiency after exposure to gamma radiation. Possible radiosensitizing effects by AT13387 treatment were evaluated through synergy analysis in monolayer and spheroid culture models. *Ex vivo* immunohistochemistry of mice xenograft tumors was performed after AT13387 treatment to study the effect on target antigen expression in an *in vivo* setting.

## RESULTS

### AT13387 inhibits proliferation and reduces the survival rate

**Figure 1:**
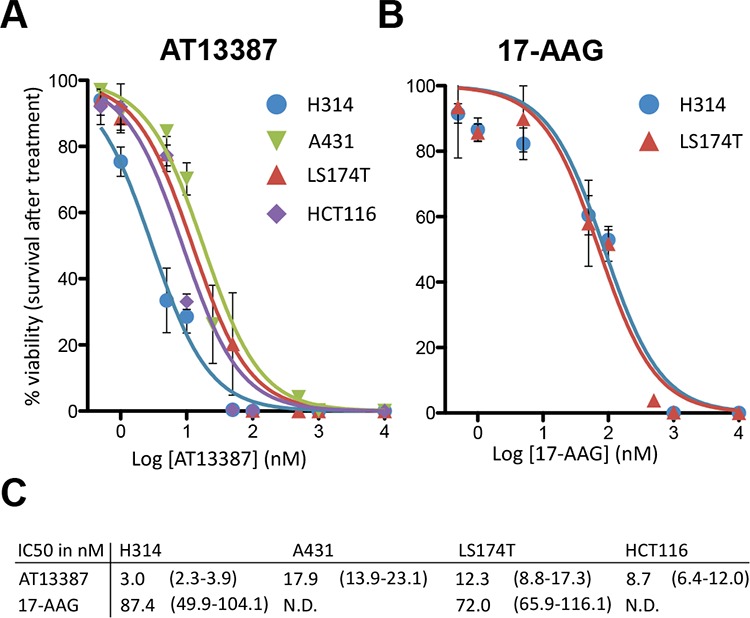
Dose response curves and IC50 analysis **A.** AT13387 treatment on H314, LS174T, A431 and HCT116 cells and **B.** 17-AAG treatment on H314 and LS174T cells. 7–21 days after drug exposure, colonies of more than 50 cells were counted. The error bars represent the standard deviation (*n* ≥ 4–8). **C.** Summary of the IC50 values (in nM) of the investigated cell lines with 95% confidence interval in parenthesis.

In order to determine inhibitor potency and the effect on cell proliferation and cell survival, clonogenic assays were performed. AT13387 markedly decreased cell viability and cell proliferation in SCC and colon cancer cell lines. The IC_50_ values for A431, HCT116, LS174T and H314 cells were in the low nanomolar range: 17.9, 8.7, 12.3 and 3 nM, respectively (Figure 1A). In comparison, the IC_50_ values for LS174T and H314 treated with 17-AAG were 6 and 30 times higher with 87 and 72 nM, respectively (Figure 1B–1C).

### Low doses of AT13387 radiosensitize cancer cells in monolayer culture

**Figure 2:**
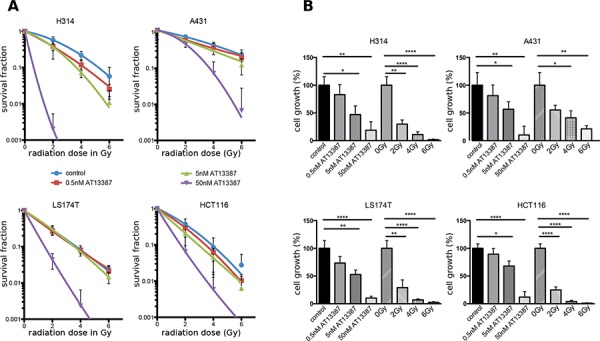
Clonogenic survival assays **A.** Dose response curves of H314, A431 LS174T, HCT116 and cells treated with AT13387 (0.5, 5, 50 nM) and radiation (2, 4 and 6 Gy). The cells were pre-plated in triplicates, incubated with AT13387 24 h later and irradiated 1 h after drug incubation. Colonies with > 50 cells were counted. The error bars represent the standard deviation (*n* ≥ 6–12). All curves are normalized to the plating efficiency of the non-irradiated controls. **B.** Effects of the AT13387 and radiation alone as measured by inhibition in cell growth (%) of the dataset in A), evaluated with Student's *t*-test with *p < 0.05, **p < 0.01, ***p < 0.001.

We determined the effect of AT13387 on radiation-induced loss of cell survival with clonogenic assays. Figure 2A shows that AT13387 affects the clonogenic survival after radiation treatment in a concentration dependent manner. The effect of the single treatments on the cell growth are summarized in Figure 2B. At a radiation dose of 4 Gy, 22% of H314 were able to grow into a colony, while combination treatment with 0.5 nM AT13387 reduced the survival by a factor of 2, to 11%. At the same radiation dose 14% of H314 cells treated 50 nM 17-AAG survived the treatment (Supplementary Figure 1A). 40% of A431 cells survived a radiation dose of 4 Gy while only 33% survived 4 Gy and 0.5 nM AT13387. At a radiation dose of 6 Gy, 0.5 nM AT13387 reduced the survival by more than a factor of two, from 25% to 12%. AT13387 treatment sensitized cells at lower concentrations than treatment with 17-AAG (Supplementary Figure 1B). Here drug doses above 50 nM were needed to radiosensitize the investigated cell lines. Analysis of the clonogenic survival data using the synergy model described by Valeriote et al. [28] displayed significantly reduced survival after irradiation and various concentrations of AT13387. When comparing survival fractions from combination treatment with calculated expected survival fractions S_exp_ from single treatments, statistically significant radiosensitizing and synergistic effects could be seen on all cell lines for 50 nM AT13387 and radiation doses of 2, 4 and 6 Gy (*p* < 0.05). Very low concentrations of AT13387 (0.5–5 nM) did not radiosensitize LS174T cells (see statistical summary in supplementary Table 1). Furthermore, Chou-Talalays combination index (CI) [29] was investigated and indicated synergistic effects for 5 out of 9 drug-radiation combinations for A431 cells and 8 out of 9 drug-radiation combinations for H314 cells (CI ≤ 0.9). CI values for the treatment combinations for the colorectal cell lines LS174T and HCT116 displayed synergistic effects or strong synergistic effects for all investigated drug and radiation doses (for CI values see supplementary Table 2). The CI value was lowered to a greater extent by increasing drug dosages as compared to radiation dosages, indicating that AT13387 potentiates the effects of radiation.

### AT13387 radiosensitizes cancer cells in tumor like conditions

**Figure 3:**
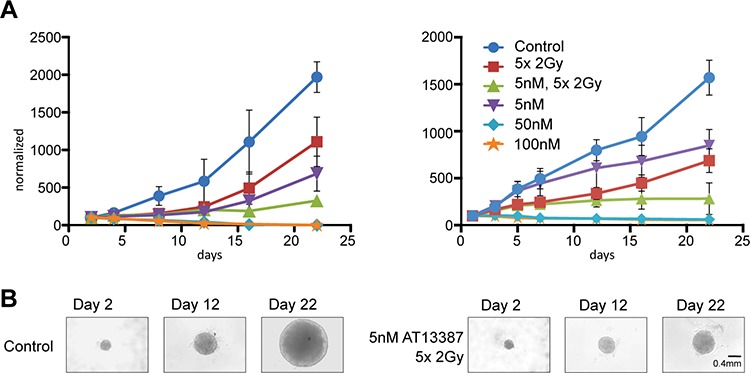
Multicellular tumor spheroid growth **A.** H314 spheroids and HCT116 spheroids treated with AT13387 (5 nM, 50 nM, 100 nM), 5 times 2 Gy radiation fractions and combination treatment of 5 nM AT13387 and radiation. 1000–3000 cells were pre-plated in an agarose coated 96-well plates, incubated with AT13387 after 24 h and irradiated 1 h after drug incubation. The error bars represent standard deviation *n* ≥ 3. All curves are normalized to the size of controls at day 1. **B.** Example of H314 spheroids after 2, 12 and 22 days.

The microenvironment and cellular organization in tumor cell spheroids has been shown to recreate that of *in vivo* tumors more closely than monolayer cell cultures. Accordingly, drug efficacy and potential radiosensitizing effects were studied in a tumor cell spheroid assay (Figure 3). Spheroid size was compared by measuring cross sectional area of a fitted ellipse at consecutive timepoints after treatment. A431 and LS174T cells were not able to form individual tumor spheroids and were excluded from analysis. After 22 days, tumor spheroids treated with a fractionated radiation dose (2 Gy/day during 5 consecutive days) grew significantly slower than controls, to about 56% of the control spheroid size (*p* < 0.05) for H314 and 57% for HCT116. H314 spheroids incubated with 5 nM AT13387 reached approximately 35% of the control size and spheroids treated with 5 nM AT13387 and fractionated radiation reached about 17%. HCT116 spheroids incubated with 5 nM AT13387 reached approximately 45% of the control size and spheroids treated with 5 nM AT13387 and fractionated radiation reached about 18%. 50 and 100 nM doses of AT13387 inhibited the growth of H314 and HCT116 spheroids and led to shrinkage and total degradation of the 3 dimensional structures. To obtain similar shrinkage and degradation with 17-AAG on H314 spheroids it was necessary to use much higher drug doses (3 × 500 nM, Supplementary Figure 2 and Figure 2A).

After 22 days a significant ( *p* < 0.0001) synergistic effect for combination treatment of 5 nM AT13387 and a fractionated radiation dose could be observed for both H314 and HCT116 spheroids.

### HSP90 inhibition may delay repair of radiation-induced DNA double strand breaks (DSB)

**Figure 4:**
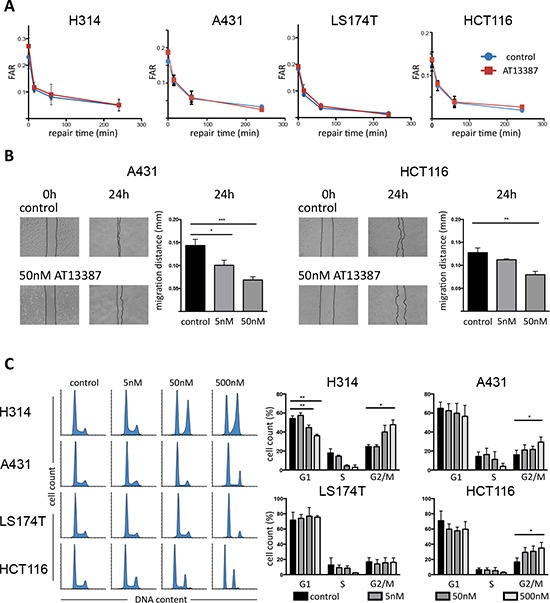
DNA DSB rejoining capacity and migration distance and cell cycle analysis **A.** PFGE analysis. H314, A431, LS174T and HCT116 cells were exposed to 200 nM AT13387 for 24 h prior to irradiation. After irradiation, cells were allowed to repair. Kinetics of DSB end rejoining was calculated by fraction of activity released (FAR) corresponding to DNA of sizes < 5.7 Mbp. The error bars represent standard deviation, *n* = 4. **B.** Cell migration assay. Left hand images represent photographs of A431 and HCT116 cultures taken at 0 h (immediately after scratching) and at 24 h with and without AT13387 treatment. The graphs show quantification of the wounded area invaded after 24 h, measured in migrated distance in mm. The error bars represent standard deviation, *n* ≥ 3. Student's *t*-test was used to calculate statistics: *p < 0.05, **p < 0.01, ***p < 0.001. **C.** Flow cytometry evaluation of cell cycle progression. Left hand histograms show representative data of DNA content as stained by DAPI/PI indicating the progression from G1 through S and to G2/M phases. Increasing concentrations of AT13387 increases the G2/M peak and depletes the S phase. Right hand side shows quantification of flow cytometry data and statistical significance from ANOVA with Tukeys post-test.

Further efforts to identify the mechanisms underlying the radiosensitizing effect of the HSP90 inhibitor AT13387 were focused on its possible impact on DNA repair mechanisms. The DSB rejoining capacity after expose of the drug was studied using pulsed-field gel electrophoresis on all four cell lines. AT13387 combined with radiation treatment induced higher amounts of DSBs than cells that were only irradiated. Contrary to expectations, AT13387 had no significant effect on the DNA repair capacity after irradiation in all four tested cell lines. Apart from a minor initial delay, treated and untreated cells showed similar repair rates after 4 h of repair time (Figure 4A). Treatment with 17-AAG reduced the repair capacity by a factor of two after 4 h of repair time (Supplementary Figure 3). Furthermore, after 24 h of repair time under normal growth conditions the difference in the DSBs repair between 17-AAG and control was still present (data not shown).

### AT13387 reduces cell migration and motility

Scratch wound healing assays were performed to assess the effects of AT13387 on cell migration and motility. The migration distance significantly decreased in concentration-depended manner after 24 h exposure to both 5 nM and 50 nM AT13387 (Figure 4B). Untreated HCT116 cells migrated 0.12 mm on average while cells treated with 50 nM moved 0.07 mm. Similarly, A431 cells treated with 50 nM AT13387 migrated only half the distance (0.07 mm) as untreated control cells (0.14 mm).

### AT13387 treatment results in G2/M phase arrest and S phase reduction

The effects of AT13387 on cell cycle progression were studied by flow cytometry. The relatively large fraction of S and G2/M phases in untreated cells indicated that all cell cultures were in exponential growth phase. A dose-dependent accumulation of cells in the G2/M phase was seen, most pronounced at the highest drug concentration in all cell lines (Figure 4C). A statistically significant increase of cells in G2/M was seen in H314, A431 and HCT116 cells. The effect was largest in H314 cells, where twice as many cells were arrested in G2 after 24 h treatment with 500 nM AT13387 compared with untreated cells. Further, AT13887 caused a reduction of cells in G1 and a reduction/depletion of S phase fraction.

### Effects of AT13387 on the protein expression *in vitro*

**Figure 5:**
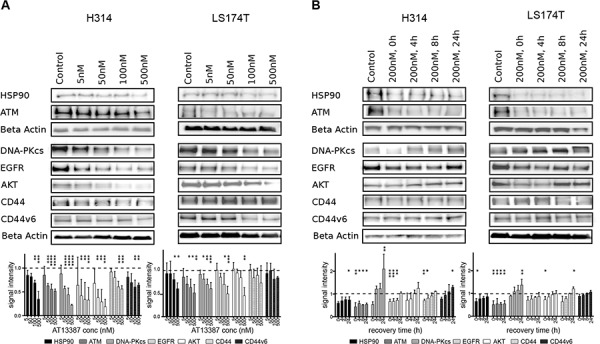
Effect of AT13387 treatment on HSP90 client protein levels (HSP90, ATM, DNA-PKcs, EGFR, AKT, CD44, CD44v6) **A.** Dose-dependent downregulation of client proteins. H314 cells and LS1474T cells treated with the indicated doses of AT13387 for 24 h. **B.** Recurrence of protein level after AT13387 treatment. H314 and LS174T cells were treated with 200 nM AT13387 for 24 h. After drug treatment cells were kept in drug-free complete medium for 0, 4, 8 and 24 h. Lysates were harvested and equivalent amounts of protein from each lysate were resolved by SDS-PAGE and immunoblotting with the indicated antibodies. The expression levels of beta actin were used to ensure equal loading. Above: Representative Western blots, Below: Western blot quantification. Protein levels were normalized to beta actin, and were normalized to the level of untreated control (dashed line). One way-ANOVA with Bonferroni post-test was used to calculate statistics: *p < 0.05, **p < 0.01, ***p < 0.001, ****p < 0.0001. Error bars represent the standard deviation, *n* = 3–6.

In order to examine molecular mechanisms of radiosensitization caused by the HSP90 inhibition, we studied the expression of several HSP90 client proteins by western blotting. We investigated the expression level of cell signaling proteins, DNA repair proteins and cell surface receptors after treatment with varying concentrations of AT13387. Treatment with 5 nM AT13387 reduced the protein level of the target HSP90, ATM, DNA-PKcs (also known as PRKDC), EGFR and AKT, an effect that was more distinct with increasing concentration (Figure 5A). After 500 nM treatment only 34% of the initial HSP90, 51% of the initial ATM, 22% of the initial DNA-PKcs level, 33% of the initial EGFR and 19% of the initial AKT level remained in H314 cells, as evaluated by ratios of signal intensity. In LS174T cells, treatment of 500 nM AT13387 reduced HSP90 expression to 58%, the client protein level to 49% for ATM, 59% for DNA-PKcs, 48% for EGFR and 44% for AKT. CD44 and CD44v6 expression did not change considerably with increasing drug concentration.

Further, to evaluate how the HSP90 inhibitor affects protein expression after removal of the drug, the recurrence of HSP90, cell surface receptors and DNA repair proteins was studied. HSP90 and ATM levels remained relatively unchanged after 4 h to 24 h of drug removal, while depletion of other HSP90 client proteins was transient. The majority of protein levels returned to normal levels after drug removal within 24 h. Figure 5B shows that detectable recovery of the proteins occurred already after 4 h under normal growth conditions. 8 h after withdrawal of the drug more than 50% of the receptors and repair proteins had recurred and after 24 h the initial protein level was reached. After 24 h DNA-PKcs protein levels were increased by 30% than before the initial treatment in LS174T cells. This effect was even greater in H314 cells.

### Effects of AT13387 on tumor size and protein expression *in vivo*

**Figure 6:**
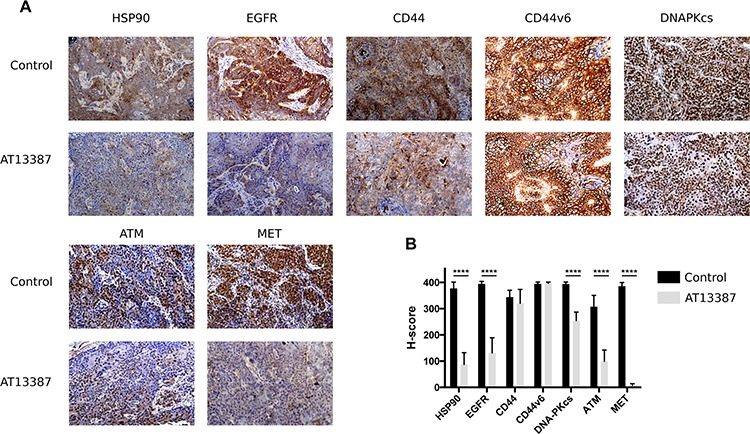
Immunohistochemical analysis on A431 tumors **A.** Representative images of *ex vivo* immunohistochemical staining for HSP90, EGFR, CD44, CD44v6, DNA-PKcs, ATM and MET expression in A431 tumor xenografts (magnification × 10). Mice in the treatment group (*n* = 6) received 5 doses of 50 mg/kg AT13387 on 5 consecutive days before dissection and analysis. The effect of AT13387 was highest on the expression pattern of HSP90, EGFR and MET. **B.** Semiquantitative analysis of immunostainings using the H-score method. Error bars represent the standard deviation, *n* = 16. One way-ANOVA with Bonferroni post-test was used to calculate statistics: *p < 0.05, **p < 0.01, ***p < 0.001, ****p < 0.0001.

To simulate a whole body environment, the efficacy of AT13387 was tested in mouse xenografts carrying EGFR and CD44v6 over-expressing A431 tumors. Mice of the treatment group were given 5 doses of 50 mg/kg AT13387 on 5 consecutive days. Tumors were removed 24 h after last injection. Animals displayed no body weight changes or other adverse effects during the treatment period. AT13387 treatment showed limited effects on tumor volume *in vivo*. The changes in tumor size were not statistically significant (data not shown), probably due to the short treatment time of 5 days. However, in the *in vivo* setting, AT13387 showed an effective and prolonged efficacy on the molecular level. As described earlier, AT13387 targets the molecular chaperon HSP90 and leads to its degradation. Our results distinctly visualize the depletion of HSP90 proteins after AT13387 treatment in the xenograft tumors (Figure 6A). The corresponding mean H-scores are depicted in Figure 6B. As evident from the figure, the expression levels of the target HSP90, the growth receptors EGFR and MET and the DNA repair proteins DNA-PKcs and ATM were significantly lower after drug treatment compared with control animals (Figure 6B). The expression of CD44 was not reduced to the same extent as HSP90, EGFR and MET, while the CD44v6 levels remained unchanged. Expression histograms are displayed in Supplementary Figure 4.

## DISCUSSION

The treatment options of advanced HNSCC and colorectal cancer are limited due to treatment resistance and severe adverse effects to healthy tissue. The concept of combination treatments that could potentiate the effects of single treatments is promising and has great potential for future therapeutic improvements. HSP90 client proteins are involved in all hallmarks of cancer, making HSP90 inhibitors promising anti-cancer drugs alone and especially in combination with other treatments. HSP90 inhibitors have been shown to block DNA damage repair in combination with radiation treatment [30, 31]. The HSP90 inhibitor NVP-AUY922 for example has been described to radiosensitize cells by depletion of proteins involved in homologous recombination resulting in mitotic entry with unresolved DNA damage [32]. A recent study indicated that AT13387 treatment could overcome resistance of melanoma cells to BRAF/MEK inhibitors. Furthermore, combination treatment of these inhibitors and AT13387 has the potential to reduce or even prevent the emergence of resistance [20]. However, clinical outcomes of first generation HSP90 inhibitors alone or in combination with other treatments has been modest due to poor solubility in water, difficulties in formulation, inconsistent pharmacokinetics and high hepatotoxicity [21]. The high-affinity HSP90 inhibitor AT13387 may overcome these limitations since it is easier to administer and may have less toxicity than the previous described drugs [24].

In this study, we investigated the efficacy of AT13387 in both 2D and 3D structures as well as mice xenografts, and tested whether treatment of AT13387 can radiosensitize SCC and colon cancer cells. We demonstrate that AT13387 induces effective cytotoxic activity in all four cancer cell lines with an IC_50_ < 20 nM, which was 6–30 times lower than for 17-AAG. For the first time, we could also demonstrate that treatment with AT13387 in vitro at clinically relevant concentrations in combination with ionizing radiation result in synergistic, radiosensitizing effects. Interestingly, the CI values, calculated by the Chou-Talalay method were lowered to a greater extent by increasing AT13387 dosages as compared with radiation dosages, indicating that AT13387 treatment potentiates the effects of radiation. Several studies have shown that drug incubation time prior irradiation is of high importance for synergy with radiation [33]. We used two different pre-incubation times, one hour and 24 hours, to establish an optimal schedule for the combination. There was no significant difference in the survival fraction of H314 cells pre-incubated 1 h or 24 h before irradiation (data not shown), both exposure times radiosensitized the cells equally. In 3D cell cultures, treatment with AT13387 significantly reduced the growth of the tumor cell spheroids. After 22 days of spheroid growth, a significant synergistic effect at 5 nM AT13387 and radiation fractions of 5 times 2 Gy was seen, and the combination effect was present at much lower dosages than with 17-AAG. The combination effect is probably higher with increasing dosages of AT13387, but at these concentrations the combined treatment effect was too great to measure since no cells survived.

We suspected that AT13387-mediated inhibition of HSP90 interrupts central factors of DSB and SSB repair, thus amplifying the radiation effect and leading to the observed synergy. Similarly, inhibition of homologous recombination in prostate and lung cancer has been reported by 17-AAG treatment. Importantly, neither the radiosensitization nor the deficient DSB repair was observed in normal fibroblasts [34]. Moreover, new client proteins of HSP90 which are essential in DSB repair pathways are constantly reported, such as ataxia-telangiectasia mutated (ATM), ataxia-telangiectasia and rad3-related (ATR), or the MRE11/RAD50/NBS1 (MRN) complex [30, 35, 36], which indicates a great potential of HSP90 inhibitors for clinical benefit. Surprisingly, even if AT13387 treatment resulted in a substantial downregulation of the essential DNA repair proteins DNA-PKcs and ATM, we found no apparent effect on the repair capacity after radiation exposure. However, radiation sensitivity is determined by a number of processes in the DNA damage response, including repair, checkpoint activation and apoptosis. Also, low levels of DNA repair proteins may be enough for a sufficient repair response but could lead to failure in check-point regulation. A recent study confirms that 80%–95% decrease of DNA-PKcs levels induced by siRNA treatment lead to extreme radiosensitivity, without affecting the DNA double strand repair [37]. Earlier results suggest that reduced levels of DNA-PKcs may lead to mitotic failure [38], an effect which is uncoupled from its direct role in the DSB repair. Further, many repair proteins have a long half-life in the cell (more than 5 days), meaning that correctly folded repair proteins available in spite of HSP90 inhibition and 24 h of drug exposure may not be enough to completely downregulate crucial proteins. These results contrast earlier studies and data for 17-AAG (Figure 4B, Supplementary Figure 3) and apparently the radiosensitization by AT13387 not only include DNA repair mechanisms, but also several cell-line-specific pathways and the destabilization and degradation of multiple HSP90 client proteins cause delays in proliferation and cell cycle impairments.

Certainly, the most lethal characteristics of an aggressive cancer phenotype are the ability to rapidly proliferate, move through tissue and form metastasis at distant locations. We could show for the first time that AT13387 treatment significantly decreases cancer cell motility and migration ability *in vitro*. Further, AT13387 disrupts cell-cycle regulation and causes G2/M arrest and simultaneous reduction of S-phase fraction. Our findings are supported by similar effects seen after treatment with other anti-HSP90 drugs, e.g. NVP-AUY922 or NVP-BEP800 [31]. These effects may be explained by the fact that cell-cycle regulators are part of the complex network of tumorigenesis, and that important factors including P53, CDK1, CDK2 and CDK4 are stabilized by HSP90 [31].

The question of oncoprotein downregulation after drug treatment has also gained great clinical attention and importance for patient response monitoring. Immunohistochemistry on A431 tumors with high expression levels of the growth factor receptor EGFR and the hyaluronan binding molecule CD44 and its splice variant CD44v6 indicated high efficacy of the drug. After treating A431 tumor bearing mice for 5 days with 50 mg/kg of AT13387 the expression of its target protein HSP90 was almost completely reduced. The same was true for the repair protein ATM and the oncogenic growth receptors EGFR and MET. Numerous studies have shown the effects of first generation HSP90 inhibitors on HER2 expression (e.g. 17-AAG, 17-DMAG) *in vitro* and *in vivo* [22, 39, 40]. HER2 is a member of the EGFR family and commonly expressed in breast, ovarian and prostate cancer. HER2 expression in HNSCC and colon cancer is insignificant and cannot be used as a biomarker for treatment outcomes using molecular imaging techniques. However, HNSCC and adenocarcinomas of the colorectal region are EGFR positive or highly positive. Our results indicate that HSP90 and EGFR could be used as biomarkers for treatment response monitoring during AT13387 therapy in HNSCC and colon cancer. Importantly, the expression of CD44v6, which is commonly overexpressed in HNSCC, was not altered as determined by IHC analysis. This makes CD44v6 a potential target for radionuclide-immunotherapy in combination with AT13387. By targeting CD44v6, patients could benefit from the radiosensitizing effects of AT13387. Another possibility would be to reduce the radiation exposure to healthy organs while maintaining therapeutic effect.

Addressing the problem of oncoprotein recurrence after drug treatment is critical for finding the optimal time window for drug and radiation treatment. Western blot results confirmed the previously described findings from *ex vivo* IHC. 24 h exposure to nanomolar concentrations of AT13387 reduced the levels of key signaling and repair proteins in a concentration dependent manner. Our results confirm recent studies on melanoma and gastrointestinal cancer cell lines, where AT13387 treatment downregulated key signaling and DNA repair proteins [4, 20, 41, 42]. The expression of the cancer stem-like cell marker CD44 and the novel HNSCC stem-like cell marker CD44v6 [43, 44] did not change considerably with increasing drug concentrations. Overexpression of CD44 is associated with increased radioresistance [45]. Yet, concentrations of 5–500 nM AT13387 could not completely downregulate the levels of the investigated proteins, suggesting that a longer exposure or higher concentrations are necessary, as was also indicated by the xenograft outcomes. Surprisingly, the tested molecules were able to recur after relatively short time intervals; 24 h after drug-removal, EGFR, AKT, CD44 and CD44v6 expression was at the same level as before treatment. The DNA-PKcs levels were even higher than before treatment, suggesting that radiation therapy would have to be initiated relatively close in time with HSP90 inhibitor therapy. These results are contrary to results where AT13387 has been described to downregulate proteins for more than 72 h [41], possibly explained by the higher concentrations used in that study. On the other hand, our *ex vivo* immunohistochemistry stains confirm effective downregulation of client proteins 24 h after the fifth daily AT13387 injection, demonstrating a long and effective biological half-life of the drug in *in vivo* environments. Furthermore, animals in the *in vivo* study displayed no body weight changes or other adverse effects during the treatment period.

Currently, AT13387 is undergoing clinical trials in phase I and II including patients with prostate carcinomas, GIST and lung cancer, but these studies do not include external beam radiation or radionuclide therapy. Our results demonstrate that AT13387 has the potential to improve radiotherapy including radio-immunotherapy outcomes significantly in squamous cell carcinomas and adenocarcinomas and restore treatment efficacy in cancers that are resistant to initial treatments.

## MATERIALS AND METHODS

### Cell culture and cell lines

The adenocarcinoma cell lines HCT116 and LS147T, obtained from the American Type Culture Collection (Manassas, VA, USA) were cultured in McCoy's 5A medium and Dulbecco's Modified Eagle Medium (DMEM), respectively, supplemented with 10% fetal bovine serum (Sigma Aldrich, St. Louis, USA), L-glutamine and antibiotics (100 IU penicillin and 100 μg/ml streptomycin) from Biochrom Kg, (Berlin, Germany). The squamous cell carcinoma cell lines A431, obtained from American Type Culture Collection (Manassas, VA, USA) and H314 from the European Collection of Cell Cultures (Salisbury, UK), were cultured in Ham's F10 and DMEM medium in a 1:1 mixture of Ham's F12, respectively, with supplements as above. Medium with supplements is referred to as “complete medium” in this article. Cells were incubated at 37°C in an atmosphere containing humidified air with 5% CO_2_. The cell lines were selected for their different radiation sensitivity and because they represent cancer types with known high HSP90 expression. The cell lines in this study were cultured for fewer than six months after purchase from the supplier.

### Tumor spheroid culture

Tumor spheroids were cultured as described earlier [46]. In short, H314, HCT116, A431 and LS174T cells were harvested by trypsinization. A defined amount of cells was seeded in 96-well, 0.15% agarose-coated culture-plate, with approximately 1000–3000 cells per well. The cells were incubated for up to 3 weeks at 37°C in an atmosphere containing humidified air with 5% CO_2_. Half of the medium was renewed every fourth to fifth day.

### Drug and radiation treatment

Drug preparation: AT13387 (Astex Pharmaceuticals, Cambridge, UK) was kindly provided by the NIH, USA. AT13387 was stored as a lyophilized powder and dissolved in 17.5% (w/v) hydroxypropyl-b-cyclodextrin before use. 17-AAG (A.G. Scientific, San Diego, CA, USA) was dissolved in DMSO and diluted in complete medium to the desired concentrations.

For *in vitro* experiments: Cells seeded for cell viability (IC_50_) and clonogenic assay were pre-plated and incubated with AT13387 (0.5, 1, 5, 15, 20, 50, 100, 500 and 1000 nM) or 17-AAG (0.5, 1, 5, 50, 100, 500 and 1000 nM). Radiation treatment was given 1 h after drug incubation using a ^137^Cs γ-ray irradiator at a dose-rate of 1 Gy/min (Best Theratronics Gammacell^®^ 40 Exactor, Springfield, USA). Spheroid cultures were grown for 1–2 days prior drug treatment. For AT13387 treatment spheroids were exposed to 5, 50 and 100 nM concentrations once. Irradiation followed 1 h after drug treatment. Half of the incubation medium was replaced with fresh complete medium every 4–5 days. For 17-AAG spheroids were either incubated with 3 × 500 nM 17-AAG or corresponding DMSO concentrations every 48 h and rinsed in complete medium 7 times before each new treatment.

For *in vivo* experiments: Animals in the treatment group (*n* = 6) were treated with AT13387 every 24 h for five consecutive days. Each time 50 mg/kg AT13387 (dissolved in 17.5% (w/v) hydroxypropyl-b-cyclodextrin) was given subcutaneously in the neck area.

### Clonogenic assay

Cell survival was performed using clonogenic assay which measures the cell colony forming ability, as described earlier [47]. In short, a defined amount of cells was pre-plated into 25 cm^2^ culture flasks with 8 ml complete medium. Cells were allowed to attach for about 24 h before incubation with complete medium containing 17-AAG or AT13387. After 25 h cells were exposed to external beam radiation. After colony formation time (H314: 21 days, A431: 14 days, HCT116: 14 days, LS174t: 7 days), cells were fixated with 95% ethanol and stained with crystal violet. Colonies of greater than 50 cells per colony were counted. Plating efficiency, PE, (number of colonies formed/number of cells seeded in the control) and the survival fraction, SF, (number of colonies formed after treatment/number of cells seeded × PE) were calculated. The curves were fitted to a linear quadratic model using Graphpad Prism 5d (Graphpad Software, San Diego, CA, USA).

### Pulsed-field gel electrophoresis (PFGE)

PFGE was performed as described previously [48]. H314 and LS174T cells were grown for two doubling times in culture medium containing 2 kBq/ml [methyl-^14^C] thymidine (Perkin Elmer), followed by irradiation with a radiation dose of 40 Gy on ice. After irradiation, cells were allowed to repair for 0, 15, 60 min and 24 h at 37°C, before trypsinization and plug formation. For the plug formation, cells were mixed with 0.6% low gelling-point agarose (InCert, BMA, Rockland, USA) at a concentration of 1.5–2.5 × 10^6^ cells/ml and transferred into plug-molds. After gelling, plugs were transferred into ESP lysis buffer at 4°C, containing 0.5 M EDTA at pH 8.0, 2% sarkosyl (N-lauroylsarcosine) (Sigma Aldrich, St. Louis, USA) and 1 mg/ml proteinase K (Roche Diagnostics, Mannheim, Germany). Plugs were transferred to HS-buffer after > 20 hours and incubated overnight (HS: High Salt; 1.85 M NaCl, 0.15 M KCl, 5 mM MgCl_2_, 2 mM EDTA, 4 mM Tris, 0.5% Triton X-100, pH 7.5) at 4°C. Before electrophoresis, plugs were washed 2 × in 0.1 M EDTA and equilibrated in 0.5 × TBE and loaded into an agarose gel (0.8% SeaKem Gold, Lonza, USA). PFGE was performed with Gene Navigator unit (Amersham Pharmacia Biotech, Uppsala, Sweden) with 120° between the fields. After electrophoresis, the gel was divided at the position of the 5.7 Mbp chromosome from *S. pombe* (BMA), and the DNA-incorporated ^14^C activity was measured by liquid scintillation. Fraction Activity Released (FAR) was calculated by dividing the fraction of radioactivity (^14^C) corresponding to DNA strands less than 5.7 Mbp and the total radioactivity in each lane.

### Cell migration and motility

The cell migration ability of A431 and HCT116 cells was studied using a scratch assay at confluence, as previously reported [49]. In short, cells were grown in 6 well plates and were washed and incubated with normal cell medium, 5 or 50 nM AT13387. Then, a narrow area on the confluent cell monolayers was scratched off with a p100 pipette tip. Images from the same scratch location were obtained directly after scratching and after 24 h incubation. Migration distance in mm was calculated as (width of the scratch at time 0–width of the scratch at 24 h)/2. Significance testing was made using two-tailed Student's *t* test and was considered significant if *p* < 0.05. Experiments were repeated three times.

### Flow cytometry

H314, A431, LS174T and HCT116 cells were incubated for 24 h with 5, 50 or 500 nM AT13387 prior to cell cycle analysis. Cells were harvested by using non-enzymatic cell dissociation solution (Sigma Aldrich, St. Louis, USA) and fixated with 70% ethanol and kept in −20°C for at least 24 h. After 2 washes with PBS cells were incubated with 10 μg/ml propidium iodide (Sigma Aldrich)/0.1% NP-40 (Sigma Aldrich) or 1 μg/ml DAPI (Sigma Aldrich) together with 5 μg/ml RNase (Sigma Aldrich) for 30 min. Cells were analyzed on a SORP BD LSRII (Becton Dickinson Biosciences, San Jose, USA) and on a Partec CyFlow Cube Sorter (Partec GmbH, Germany) with integrated FSC express version 4.0 (De Novo Software, CA, USA) software. FCS files were analyzed with Flowing Software 2.5.1 (Turku Centre for Biotechnology, Finland) and ModFit LT program (Verity Software House, Topsham, ME, USA). For the evaluation of intracellular DNA content, at least 10,000 events for each point were analyzed in at least three different experiments. Statistical analysis of the cell cycle phases was performed using a one-way ANOVA with Tukey posttest.

### Western blot

For western blot analysis, whole-cell lysates were prepared according to standard protocols. The protein concentration was determined by BCA protein assay (Pierce Biotechnology, Rockford, USA). Samples were separated on a SDS PAGE and afterwards transferred to a nitrocellulose membrane by wet blotting. The nitrocellulose membrane was blocked for 1 hour in PBS with 5% BSA and then incubated with the primary antibodies over night at 4°C. Primary antibodies used were anti-DNA-PKcs (Abcam, Cambridge, UK), anti-EGFR (Abcam, Cambridge, UK), anti-CD44 (Biolegend, San Diego, CA, USA), CD44v6 (AbD serotec, Bio-rad, UK), anti-AKT (Santa Cruz Biotechnology (Santa Cruz, CA, USA) and anti-beta-actin (Sigma-Aldrich, St. Louis, USA). After washing in PBS with 1% Tween-20, membrane was incubated with respective and species-specific Horse Radish Peroxidase-labeled secondary antibody (Invitrogen). Immunoreactive bands were visualized with a CCD camera (SuperCCD HR, Fujifilm, Japan) after treatment with electrochemiluminescent solution (Immobilon, Millipore, Bedford, USA) according to instructions from the manufacturer. Quantitative densitometric analysis of western blot results was performed using ImageJ software version 1.48 (NIH, Bethesda, MD, USA). Relative levels of protein expression were compared with the beta-actin expression from the same cell lysate. One-way ANOVA with Bonferroni post-test was used to evaluate significant differences in signal intensity.

### *In vivo* studies

All experiments complied with the current Swedish law and were performed with permission granted by the Uppsala Committee of Animal Research Ethics. The nu/nu Balb/c mice (female; *n* = 12) were housed in a controlled environment and fed ad libitum. Tumor xenografts were formed by subcutaneous inoculation of approximately 9 × 10^6^ A431 cells (high EGFR and CD44v6 expression) suspended in 150 μl 1:1 cell medium : matrigel in the right posterior leg.

After tumor growth of 2 weeks (animal age of 8 weeks), animals of the treatment group (*n* = 6) were treated with AT13387 (see drug and radiation treatment). All animals were euthanized 24 h after last injection dose with a mixture of ketamine and zylazine followed by heart puncture. The tumors were removed and directly fixated in formalin. The average animal weight was 16.1 ± 1.5 g, and average tumor weight was 0.41 ± 0.036 (SEM) g.

### Immunohistochemistry

A431 tumors were fixated in formalin directly after removal. Next, tumors were paraffin-embedded, sectioned, and deparaffinised. Antigen retrieval was achieved by microwaving (10 + 15 min) in citrate buffer (DAKO, S2369) or Tris-EDTA buffer (DAKO, S2367). Immunostaining with anti-HSP90 (Abcam, UK), anti-DNA-PKcs (Abcam, UK), anti-CD44 (Abcam, UK), anti-CD44v6 (AbD serotec), anti-EGFR, anti-MET and anti-ATM (Abcam, UK) performed according to manufactures instructions. The secondary step has been Dako EnVision+ System-HRP labeled polymer anti-rabbit or EnVision FLEX/HRP (K8000, Dako). The sections were counterstained with Mayer's hematoxylin (DAKO).

### Image analysis

Images of cell spheroids (magnification × 4), migration (magnification × 4) and IHC staining (magnification × 10) were obtained using a Nikon D3000 digital camera mounted on an inverted Nikon Diaphot-TMD microscope. Spheroids were photographed every second to every fifth day for about 3 weeks. The spheroid images were analyzed semi-automatically using the MTS Research Analysis Tool, RM Medic-Tech (Uppsala, Sweden) and manually with the ImageJ software version 1.48 (NIH, Bethesda, MD, USA). Outlines of the spheroid shape were drawn and the area of the aggregates was measured. The migration pictures were analyzed with ImageJ version 1.48 (NIH, Bethesda, MD, USA).

Immunohistochemistry assays were semi-quantified according to the H-score method as presented earlier [50, 51]. Briefly, the H-score is acquired by manual scoring of each cell in 5 intensity groups, 0 = no staining, 1 = weak staining, 2 = moderate staining, 3 = dark staining, 4 = maximum staining. The H-score is then the sum of (0*p0)+(1*p1)+(2*p2)+(3*p3)+(4*p 4), where *p*0, 1, 2, 3, 4 is the percentage cells in the corresponding group, yielding a H-score range of 0–400. H-scoring was done on 16 separate sections of each tumor tissue sample, counting 100 cells per section for a total of 1600 scored cells per sample. The immunohistochemistry assays were scored blinded with respect to target and treatment. One-way ANOVA with Bonferroni post-test was used to evaluate significant differences in H-score.

### Statistical analysis and curve fitting

Microsoft Office Excel 2008 for Mac (Microsoft, Redmond, WA, USA) and GraphPad Prism 5d for Mac (GraphPad Software, San Diego, USA) were used for data processing, graph plotting and statistical analysis. For statistical analysis of cell survival after treatment with drug, treatment outcome was compared between single treatment and combination treatment using the additive model of survival fractions described by Valeriote et al. [28] and using the Chou-Talalay method [29]. Briefly, the additive model assumes that a pure additive effect will lead to an expected combination survival fraction S_exp_ = S_drug_ * S_radiation_. If the observed combination survival fraction S_obs_ is significantly lower than S_exp_ as determined by Student's *t*-test, the combination treatment is regarded as synergistic. If S_obs_ was not significantly different from S_exp_, but still lower than S_radiation_ or S_drug_, the combination was regarded as additive, whereas a significantly higher S_obs_ than S_exp_, S_drug_ and S_radiation_ was regarded as antagonistic. We calculated the combination index (CI) according to the Chou-Talalay method (non-constant rate ratio) using the CompuSyn 3.0.1 software (CompuSyn, Inc., New York, USA). (CI > 1.1, antagonism; 0.9 > CI ≤ 1.1, additive effect; 0.2 > CI ≤ 0.9, synergism; CI < 0.2 strong synergism). In the case of multicellular spheroids the synergy model described by Valeriote et al. [28] was used (see analysis for clonogenic survival data). In these experiments tumor volume changes replaced the survival fraction.

For the IC_50_ analysis and curve fit, a normalized log(response) inhibition model was used, with a fixed Hill slope of −1: Y = 100/(1+10^((X-LogIC_50_))). Where Y = survival (percentage), X = drug concentration, IC_50_ = drug concentration at 50% survival. No parameter constraints were used. Significance testing of plating efficiencies was made using two-tailed Student's *t* test and was considered significant if *p* < 0.05.

## CONCLUSION

We could for the first time demonstrate potent anti-tumor effects of the novel HSP90 inhibitor AT13387 alone and in combination with radiation in SCC and colon cancer cells *in vitro* as well as excellent efficacy on HSP90 client protein expression *in vivo*. Especially the synergistic combination effects even at low doses AT13387 are promising for either radiation dose reduction and minimization of side effects, or increased therapeutic response. The mechanism behind this effect is likely to relate to the observed downregulation of DNA repair, cell signaling and cell growth oncoproteins after AT13387 treatment. These results strengthen the case for further clinical studies of HSP90 inhibitors and radiation therapy co-treatment.

## SUPPLEMENTARY FIGURES AND TABLES



## Abbreviations

HSP90: Heat shock protein 90
SCC: squamous cell carcinoma
HN: head and neck
AT13387: (2,4-dihydroxy-5-isopropyl-phenyl)-[5-(4-methyl-piperazin-1-ylmethyl)-1,3-dihydro-isoindol-2-yl] thanone, l-lactic acid salt)
17-AAG: 17-N-Allylamino-17-demethoxygeldanamycin
GIST: gastrointestinal stromal tumors
5-FU: 5-Fluorouracil
CI: Chou-Talalays combination index
DMEM: Dulbecco's Modified Eagle Medium
PFGE: Pulsed-field gel electrophoresis
DSB: DNA double strand break
FAR: fraction of activity released
PE: plating efficiency
SF: survival fraction
EGFR: epidermal growth factor receptor
DNA-PKcs: DNA dependent protein kinase-catalytic subunit
17-DMAG: 17-(dimethylaminoethylamino)-17-demethoxygeldanamycin
ATM: ataxia-telangiectasia mutated
ATR: ataxia-telangiectasia and rad3-related
MRN: MRE11/RAD50/NBS1

## References

[1] Kabakov A, Kudryavtsev V, Gabai V (2010). Hsp90 inhibitors as promising agents for radiotherapy. Journal of molecular medicine (Berlin, Germany).

[2] Katz D, Ito E, Liu F-F (2009). On the path to seeking novel radiosensitizers. International journal of radiation oncology, biology, physics.

[3] Kunkler IH, Audisio R, Belkacemi Y, Betz M, Gore E, Hoffe S, Kirova Y, Koper P, Lagrange JLL, Markouizou A, Pfeffer R, Villa S, Force SRT (2014). Review of current best practice and priorities for research in radiation oncology for elderly patients with cancer: the International Society of Geriatric Oncology (SIOG) task force. Annals of oncology: official journal of the European Society for Medical Oncology / ESMO.

[4] Hong D, Banerji U, Tavana B, George G, Aaron J, Kurzrock R (2013). Targeting the molecular chaperone heat shock protein 90 (HSP90): lessons learned and future directions. Cancer treatment reviews.

[5] Den R, Lu B (2012). Heat shock protein 90 inhibition: rationale and clinical potential. Therapeutic advances in medical oncology.

[6] Huang W, Wu Q, Zhang M, Kong Y, Cao P, Zheng W (2015). Novel Hsp90 inhibitor FW-04–806 displays potent antitumor effects in HER2-positive breast cancer cells as a single agent or in combination with lapatinib. Cancer letters.

[7] Lu X, Xiao L, Wang L, Ruden DM (2012). Hsp90 inhibitors and drug resistance in cancer: the potential benefits of combination therapies of Hsp90 inhibitors and other anti-cancer drugs. Biochem Pharmacol.

[8] Chehab M, Caza T, Skotnicki K, Landas S, Bratslavsky G, Mollapour M, Bourboulia D (2015). Targeting Hsp90 in urothelial carcinoma. Oncotarget.

[9] Cohen SM, Mukerji R, Samadi AK, Zhang X, Zhao H, Blagg BS, Cohen MS (2012). Novel C-terminal Hsp90 inhibitor for head and neck squamous cell cancer (HNSCC) with *in vivo* efficacy and improved toxicity profiles compared with standard agents. Annals of surgical oncology.

[10] Tsutsumi S, Beebe K, Neckers L (2009). Impact of heat-shock protein 90 on cancer metastasis. Future oncology (London, England).

[11] Drecoll E, Nitsche U, Bauer K, Berezowska S, Slotta-Huspenina J, Rosenberg R, Langer R (2014). Expression analysis of heat shock protein 90 (HSP90) and Her2 in colon carcinoma. International journal of colorectal disease.

[12] Jego G, Hazoumé A, Seigneuric R, Garrido C (2013). Targeting heat shock proteins in cancer. Cancer letters.

[13] Nagaraju GP, Alese OB, Landry J, Diaz R, El-Rayes BF (2014). HSP90 inhibition downregulates thymidylate synthase and sensitizes colorectal cancer cell lines to the effect of 5FU-based chemotherapy. Oncotarget.

[14] Misso G, Giuberti G, Lombardi A, Grimaldi A, Ricciardiello F, Giordano A, Tagliaferri P, Abbruzzese A, Caraglia M (2013). Pharmacological inhibition of HSP90 and ras activity as a new strategy in the treatment of HNSCC. Journal of Cellular Physiology.

[15] Budillon A, Bruzzese F, Di Gennaro E, Caraglia M (2005). Multiple-target drugs: inhibitors of heat shock protein 90 and of histone deacetylase. Current Drug Targets.

[16] Koga F, Kihara K, Neckers L (2009). Inhibition of cancer invasion and metastasis by targeting the molecular chaperone heat-shock protein 90. Anticancer research.

[17] Ahsan A, Ramanand S, Whitehead C, Hiniker S, Rehemtulla A, Pratt W, Jolly S, Gouveia C, Truong K, Van Waes C, Ray D, Lawrence T, Nyati M (2012). Wild-type EGFR is stabilized by direct interaction with HSP90 in cancer cells and tumors. Neoplasia.

[18] Jiao Y, Ou W, Meng F, Zhou H, Wang A (2011). Targeting HSP90 in ovarian cancers with multiple receptor tyrosine kinase coactivation. Molecular cancer.

[19] Ambati SR, Lopes E, Kosugi K, Mony U, Zehir A, Shah SK, Taldone T, Moreira AL, Meyers PA, Chiosis G (2014). Pre-clinical efficacy of PU-H71, a novel HSP90 inhibitor, alone and in combination with bortezomib in Ewing sarcoma. Molecular oncology.

[20] Smyth T, Paraiso KH, Hearn K, Rodriguez-Lopez AM, Munck JM, Haarberg HE, Sondak VK, Thompson NT, Azab M, Lyons JF, Smalley KS, Wallis NG (2014). Inhibition of HSP90 by AT387 Delays the Emergence of Resistance to BRAF Inhibitors and Overcomes Resistance to Dual BRAF and MEK Inhibition in Melanoma Models. Molecular cancer therapeutics.

[21] Samuni Y, Ishii H, Hyodo F, Samuni U, Krishna M, Goldstein S, Mitchell J (2010). Reactive oxygen species mediate hepatotoxicity induced by the Hsp90 inhibitor geldanamycin and its analogs. Free radical biology & medicine.

[22] Shao Y, Wang B, Shi D, Miao S, Manivel P, Krishna R (2014). Synuclein gamma protects HER2 and renders resistance to Hsp90 disruption. Molecular Oncology.

[23] Woodhead AJ, Angove H, Carr MG, Chessari G, Congreve M, Coyle JE, Cosme J, Graham B, Day PJ, Downham R, Fazal L, Feltell R, Figueroa E, Frederickson M, Lewis J, McMenamin R (2010). Discovery of (2,4-dihydroxy-5-isopropylphenyl)-[5-(4-methylpiperazin-1-ylmethyl)-1,3-dihydrois oindol-2-yl]methanone (AT13387), a novel inhibitor of the molecular chaperone Hsp90 by fragment based drug design. J Med Chem.

[24] Kang M, Reynolds C, Houghton P, Alexander D, Morton C, Kolb E, Gorlick R, Keir S, Carol H, Lock R, Maris J, Wozniak A, Smith M (2012). Initial testing (Stage 1) of AT13387, an HSP90 inhibitor, by the pediatric preclinical testing program. Pediatric blood & cancer.

[25] Shapiro GI, Kwak E, Dezube BJ, Yule M, Ayrton J, Lyons J, Mahadevan D (2015). First-in-Human Phase I Dose Escalation Study of a Second-Generation Non-Ansamycin HSP90 Inhibitor, AT13387, in Patients with Advanced Solid Tumors. Clinical cancer research: an official journal of the American Association for Cancer Research.

[26] Rothenberg SM, Ellisen LW (2012). The molecular pathogenesis of head and neck squamous cell carcinoma. The Journal of Clinical Investigation.

[27] Chien CY, Tsai HT, Su LJ, Chuang HC, Shiu LY, Huang CC, Fang FM, Yu CC, Su H-T, Chen CH (2014). Aurora-A signaling is activated in advanced stage of squamous cell carcinoma of head and neck cancer and requires osteopontin to stimulate invasive behavior. Oncotarget.

[28] Valeriote F, Lin H (1975). Synergistic interaction of anticancer agents: a cellular perspective. Cancer chemotherapy reports Part 1.

[29] Chou TC (2010). Drug combination studies and their synergy quantification using the Chou-Talalay method. Cancer Res.

[30] Dote H, Burgan W, Camphausen K, Tofilon P (2006). Inhibition of hsp90 compromises the DNA damage response to radiation. Cancer research.

[31] Stingl L, Stuhmer T, Chatterjee M, Jensen MR, Flentje M, Djuzenova CS (2010). Novel HSP90 inhibitors, NVP-AUY922 and NVP-BEP800, radiosensitise tumour cells through cell-cycle impairment, increased DNA damage and repair protraction. Br J Cancer.

[32] Zaidi S, McLaughlin M, Bhide S, Eccles S, Workman P, Nutting C, Huddart R, Harrington K (2012). The HSP90 inhibitor NVP-AUY922 radiosensitizes by abrogation of homologous recombination resulting in mitotic entry with unresolved DNA damage. PloS one.

[33] Koll T, Feis S, Wright M, Teniola M, Richardson M, Robles A, Bradsher J, Capala J, Varticovski L (2008). HSP90 inhibitor, DMAG, synergizes with radiation of lung cancer cells by interfering with base excision and ATM-mediated DNA repair. Molecular cancer therapeutics.

[34] Noguchi M, Yu D, Hirayama R, Ninomiya Y, Sekine E, Kubota N, Ando K, Okayasu R (2006). Inhibition of homologous recombination repair in irradiated tumor cells pretreated with Hsp90 inhibitor 17-allylamino-17-demethoxygeldanamycin. Biochemical and biophysical research communications.

[35] Kim Y-M, Pyo H (2012). Cooperative enhancement of radiosensitivity after combined treatment of 17-(allylamino)-17-demethoxygeldanamycin and celecoxib in human lung and colon cancer cell lines. DNA and cell biology.

[36] Niewidok N, Wack L-J, Schiessl S, Stingl L, Katzer A, Polat B, Sukhorukov V, Flentje M, Djuzenova C (2012). Hsp90 Inhibitors NVP-AUY922 and NVP-BEP800 May Exert a Significant Radiosensitization on Tumor Cells along with a Cell Type-Specific Cytotoxicity. Translational oncology.

[37] Gustafsson A-S, Abramenkovs A, Stenerlöw B (2014). Suppression of DNA-dependent protein kinase sensitize cells to radiation without affecting DSB repair. Mutation Research/Fundamental and Molecular Mechanisms of Mutagenesis.

[38] Shang Z-F, Huang B, Xu Q-Z, Zhang S-M, Fan R, Liu X-D, Wang Y, Zhou P-K (2010). Inactivation of DNA-dependent protein kinase leads to spindle disruption and mitotic catastrophe with attenuated checkpoint protein 2 Phosphorylation in response to DNA damage. Cancer research.

[39] van de Ven SM, Elias SG, Chan CT, Miao Z, Cheng Z, De A, Gambhir SS (2012). Optical imaging with her2-targeted affibody molecules can monitor hsp90 treatment response in a breast cancer xenograft mouse model. Clinical cancer research : an official journal of the American Association for Cancer Research.

[40] Oude Munnink TH, de Vries EG, Vedelaar SR, Timmer-Bosscha H, Schröder CP, Brouwers AH, Lub-de Hooge MN (2012). Lapatinib and 17AAG reduce 8Zr-trastuzumab-F(ab’)2 uptake in SKBR3 tumor xenografts. Molecular pharmaceutics.

[41] Graham B, Curry J, Smyth T, Fazal L, Feltell R, Harada I, Coyle J, Williams B, Reule M, Angove H, Cross D, Lyons J, Wallis N, Thompson N (2012). The heat shock protein 90 inhibitor, AT13387, displays a long duration of action *in vitro* and *in vivo* in non-small cell lung cancer. Cancer science.

[42] Smyth T, Van Looy T, Curry J, Rodriguez-Lopez A, Wozniak A, Zhu M, Donsky R, Morgan J, Mayeda M, Fletcher J, Schöffski P, Lyons J, Thompson N, Wallis N (2012). The HSP90 inhibitor, AT13387, is effective against imatinib-sensitive and -resistant gastrointestinal stromal tumor models. Molecular cancer therapeutics.

[43] Spiegelberg D, Kuku G, Selvaraju R, Nestor M (2014). Characterization of CD44 variant expression in head and neck squamous cell carcinomas. Tumour Biol.

[44] Haylock AK, Spiegelberg D, Nilvebrant J, Sandstrom K, Nestor M (2014). *In vivo* characterization of the novel CDv6-targeting Fab fragment AbD15179 for molecular imaging of squamous cell carcinoma: a dual-isotope study. EJNMMI Res.

[45] Sahlberg SH, Spiegelberg D, Glimelius B, Stenerlow B, Nestor M (2014). Evaluation of cancer stem cell markers CD133, CD44, CD24: association with AKT isoforms and radiation resistance in colon cancer cells. PLoS One.

[46] Friedrich J, Seidel C, Ebner R, Kunz-Schughart L (2009). Spheroid-based drug screen: considerations and practical approach. Nature protocols.

[47] Franken N, Rodermond H, Stap J, Haveman J, van Bree C (2006). Clonogenic assay of cells *in vitro*. Nature protocols.

[48] Karlsson KH, Radulescu I, Rydberg B, Stenerlow B (2008). Repair of radiation-induced heat-labile sites is independent of DNA-PKcs, XRCC1 and PARP. Radiation research.

[49] Liang C-CC, Park AY, Guan J-LL (2007). *In vitro* scratch assay: a convenient and inexpensive method for analysis of cell migration *in vitro*. Nature protocols.

[50] Cappuzzo F, Hirsch FR, Rossi E, Bartolini S, Ceresoli GL, Bemis L, Haney J, Witta S, Danenberg K, Domenichini I, Ludovini V, Magrini E, Gregorc V, Doglioni C, Sidoni A, Tonato M (2005). Epidermal Growth Factor Receptor Gene and Protein and Gefitinib Sensitivity in Non-Small-Cell Lung Cancer. Journal of the National Cancer Institute.

[51] Hirsch FR, Varella-Garcia M, Bunn PA, Di Maria MV, Veve R, Bremnes RM, Barón AE, Zeng C, Franklin WA (2003). Epidermal Growth Factor Receptor in Non-Small-Cell Lung Carcinomas: Correlation Between Gene Copy Number and Protein Expression and Impact on Prognosis. Journal of Clinical Oncology.

