# Supplementary material for: The novel HSP90 inhibitor AT13387 potentiates radiation effects in squamous cell carcinoma and adenocarcinoma cells
Source: Oncotarget. 2015 Oct 6;6(34):35652–66. doi: 10.18632/oncotarget.5363 (PMC4742132; doi:10.18632/oncotarget.5363)
Supplement: Supplementary file 1 [file oncotarget-06-35652-s001.pdf]

SUPPLEMENTARY FIGURES AND TABLES

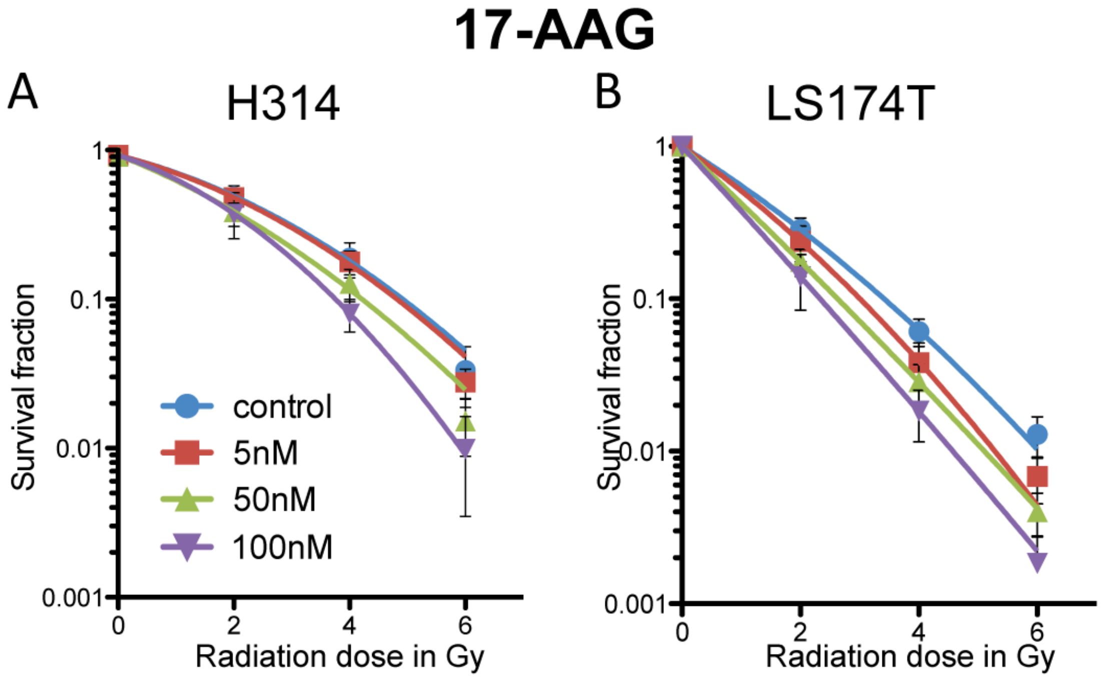

Supplementary Figure S1: Clonogenic survival assays. A. H314 and **B.** LS174T cells treated with 17-AAG (5, 50, 100 nM) and radiation (2, 4 and 6 Gy). The cells were pre-plated in triplicates, incubated with AT13387 24 h later and irradiated 1 h after drug incubation. Colonies with > 50 cells were counted. The error bars represent the standard deviation, $n \geq 6$–12. All curves are normalized to the plating efficiency of the non-irradiated controls.

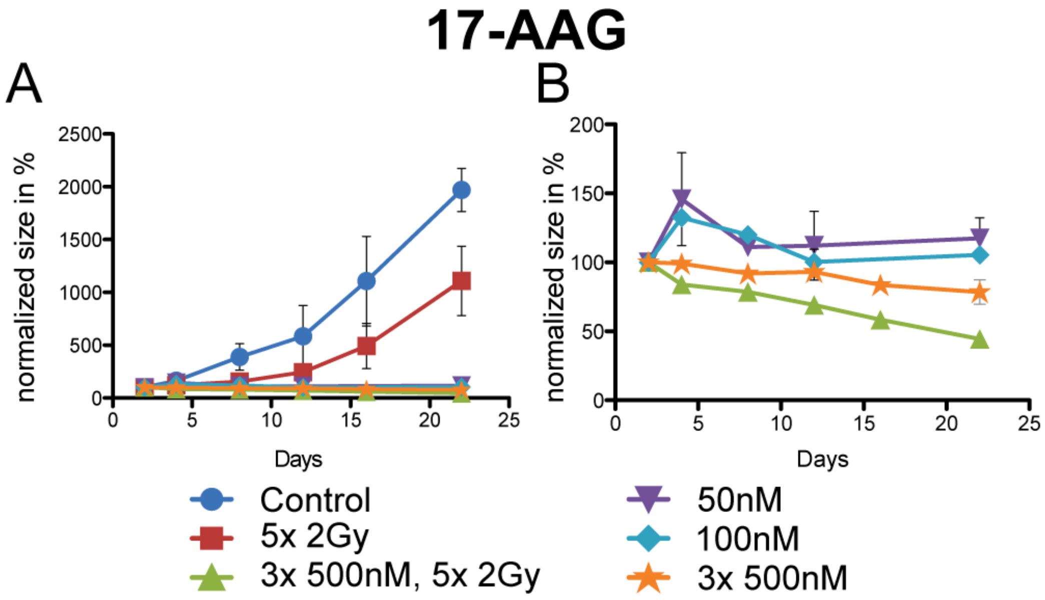

Supplementary Figure S2: Multicellular tumor spheroid growth. A. H314 cells treated with 17-AAG (50 nM, 100 nM and 3 times 500 nM, see Materials and Methods for details), 5 times 2 Gy radiation fractions and combination treatment of 3 times 500 nM 17-AAG and 5 times 2 Gy. **B.** The right-hand side graph is a closer view of the left-hand side, to better show differences in the low-growth region. 1000–3000 cells were pre-plated in an agarose coated 96-well plates, incubated with 17-AAG after 24 h and irradiated 1 h after drug incubation. The error bars represent standard deviation, $n \geq 3$. All curves are normalized to the size of controls at day 1.

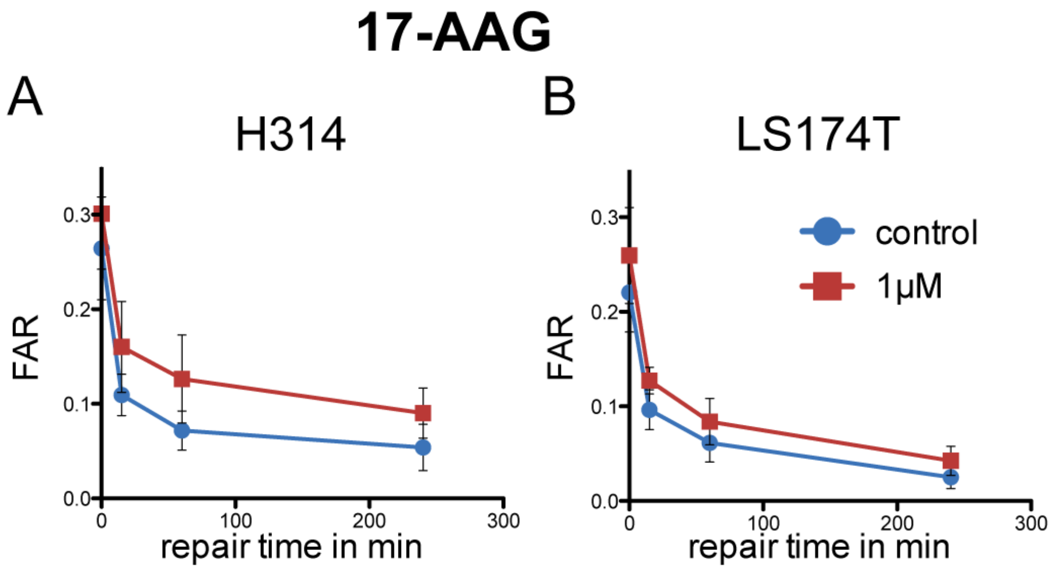

Supplementary Figure S3: DNA DSB rejoining after irradiation and treatment of HSP90 inhibitors measured by PFGE. A. H314 and **B.** LS174T cells exposed to 1 µM 17-AAG for 24 h prior radiation. After irradiation, cells were allowed to repair. Kinetics of DSB end rejoining was calculated by fraction of activity released (FAR) corresponding to DNA of sizes < 5.7 Mbp. The error bars represent the standard deviation, $n = 4$.

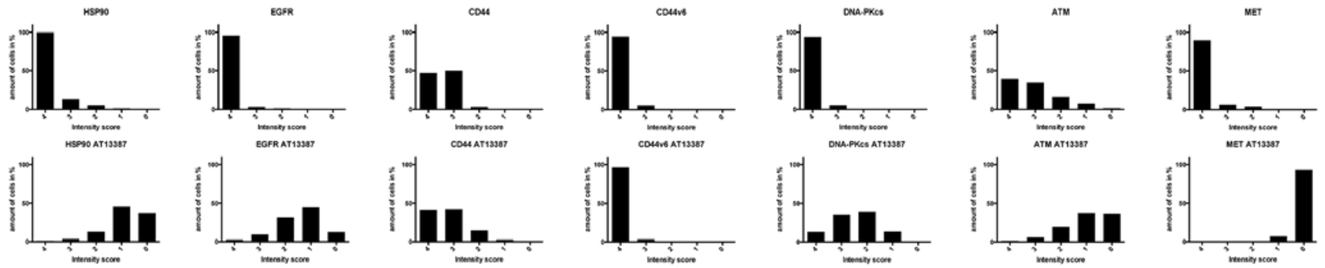

Supplementary Figure S4: Histogram of IHC intensity distribution. Scoring intensity of HSP90, EGFR, CD44, CD44v6, DNA-Pkcs, ATM and MET immunohistochemistry stainings. 0 = no staining, 1 = weak staining, 2 = moderate staining, 3 = dark staining, 4 = maximum staining.

Supplementary Table S1: Statistical evaluation of synergy, additivity or antagonism of combined AT13387 and external beam radiation treatment using the synergy model as described by Valeriote [28]

AT13387 in nM	Radiation dose in Gy	Statistical evaluation of synergy			
		H314	A431	LS174T	HCT116
0.5	2	****	Additive	Additive	Additive
0.5	4	****	Additive	Additive	Additive
0.5	6	****	Additive	Additive	**
5	2	**	*	Additive	Additive
5	4	****	*	Additive	Additive
5	6	*	**	Additive	****
50	2	*	–	*	–
50	4	–	–	*	–
50	6	–	–	***	–

Survival fractions of combination treatment were compared to expected combination survival calculated from individual treatments using Student's *t*-test.
Additive = no significant difference. Antagonism = significantly higher survival of combination treatment, - no cell survival. For synergy, *, **, *** and **** significant $p < 0.05$, 0.01, 0.001 and 0.0001, respectively.

Supplementary Table S2: Combination index (CI) value of the combination therapy of AT13387 and radiation treatment in H314, A431, LS174T and HCT116 cells

AT13387 in nM	Radiation dose in Gy	Combination Index			
		H314	A431	LS174T	HCT116
0.5	2	0.77	0.85	0.27	0.30
0.5	4	0.84	1.02	0.08	0.06
0.5	6	0.69	0.86	0.02	0.01
5	2	1.15	1.85	0.30	0.21
5	4	0.71	1.26	0.07	0.04
5	6	0.51	0.72	0.01	0.01
50	2	0.09	8.41	0.04	0.06
50	4	0.09	0.72	2.34E–03	5.88E–03
50	6	2.19E–03	0.14	8.77E–04	1.00E–13

(CI > 1.1, antagonism; 0.9 > CI ≤ 1.1, additive effect; 0.2 > CI ≤ 0.9, synergism; CI < 0.2 strong synergism).
